# Effect of supplemental methyl sulfonyl methane on performance, carcass and meat quality and oxidative status in chronic cyclic heat-stressed finishing broilers

**DOI:** 10.1016/j.psj.2022.102321

**Published:** 2022-11-09

**Authors:** Huaiyong Zhang, Maryam Majdeddin, Jeroen Degroote, Elout Van Liefferinge, Noémie Van Noten, Céline Van Kerschaver, Mario Vandaele, Juliano Cesar De Paula Dorigam, Joris Michiels

**Affiliations:** ⁎ Laboratory for Animal Nutrition and Animal Product Quality, Department of Animal Sciences and Aquatic Ecology, Ghent University, Ghent 9000, Belgium; † Evonik Operations GmbH, 63457 Hanau-Wolfgang, Germany

**Keywords:** antioxidant capacity, broiler, heat stress, methyl sulfonyl methane, performance

## Abstract

Methyl sulfonyl methane (**MSM**) is available as a dietary supplement for human and has been associated with multiple health benefits such as reduction of oxidative stress. Heat stress (**HS**) is an environmental stressor challenging poultry production and known to inflict oxidative stress. We hypothesized that dietary MSM could attenuate HS-induced detrimental effects in broilers mediated by enhancement of antioxidant defenses. Hence, seven hundred ninety-two 1-day-old male Ross 308 broilers were allocated to 3 dietary treatments composed of corn-soybean meal diets with 0 (**Ctrl**), 1, or 2 g/kg MSM, with 12 replicates (22 birds each) per treatment for 39 d and subjected to a chronic cyclic HS model (temperature of 34°C and 52–58% relative humidity for 6 h daily) from d 24 to 39. MSM at 1 and 2 g/kg linearly increased daily gain and decreased feed-to-gain ratio compared with Ctrl in the grower phase (d 10–21, both *P* < 0.05). In the finisher phase (d 21–39) none of the performance and carcass indices were affected by treatment (*P* > 0.05). Nonetheless, data suggest reduced mortality by feeding MSM during HS. Also, during HS the diets with graded levels of MSM resulted in reduced rectal temperatures (*P* < 0.05) along with linearly decreased panting frequency on d 24 (*P* < 0.05). MSM supplemented birds showed a trend for linearly decreased thiobarbituric acid reactive substances of breast meat upon simulated retail display (*P* = 0.078). In addition, MSM administration linearly decreased lipid oxidation in plasma (d 25 and 39, *P* < 0.05) and breast muscle at d 23 (*P* < 0.05), concomitantly with linearly increased glutathione levels in erythrocytes (d 23 and 39, *P* < 0.05; d 25, *P* < 0.1) and breast muscle (d 23, *P* < 0.05; d 39, *P* < 0.1). In conclusion, MSM increased growth performance of broilers during grower phase, and exhibited positive effects on heat tolerance mediated by improved antioxidant capacity in broilers resulting in lower mortality in finisher phase.

## INTRODUCTION

Heat stress (**HS**) is a vital environmental stressor challenging poultry production in subtropical and tropical countries and during heat waves in regions with milder climates. The covering of feathers, absence of sweat glands, and high metabolic rate of modern strains make broilers very susceptible to high temperatures. An obvious consequence is that HS reduces feed intake (**FI**) and nutrient absorption (Wiernusz and Teeter, 1996; Habashy et al., 2017). HS also causes many adverse physiological alterations such as suppressed immunocompetence, oxidative stress, endocrine disorders, leg abnormalities, and muscle tremors (Tao and Xin, 2003; Renaudeau et al., 2012; Akbarian et al., 2016). These physiological disorders accentuate the reduction in FI, and furthermore reduce feed efficiency, increase mortality, and impair animal welfare and productivity. Of note, high ambient temperatures could increase the production of reactive oxygen species (**ROS**), which causes damage to critical biomolecules including lipids, proteins, and DNA and disturbs redox homeostasis, resulting in reduced meat quality, increased tissue damage, and bone loss (Akbarian et al., 2016). For instance, results from our recent study show that HS decreased superoxide dismutase (**SOD**) activity in ileal mucosa, as well as induced a higher concentration of thiobarbituric acid reactive substances (**TBARS**) expressed as malondialdehyde (**MDA**), a product of polyunsaturated fatty acids’ peroxidation, in the ileal mucosa and plasma of chronic cyclic heat-stressed broilers (Zhang et al., 2021). Meanwhile, compared to the control and pair-fed group, chronic HS increased the secretion of inflammatory mediators such as interleukin 6 (**IL-6**), IL-1β, and tumor necrosis factor α (**TNF-α**) (Zhang et al., 2021). Therefore, it is pressing to seek effective treatment programs to ameliorate the detrimental effects of HS in broilers. In practice, implementing environmental mitigation strategies is prioritized including lowering stocking densities, increasing the ventilation rate and application of pad/spray cooling systems, and using different flooring systems (Renaudeau et al., 2012; Abo Ghanima et al., 2020). Moreover, several nutritional approaches such as supplementation of diets with vitamins (Zhang et al., 2021), probiotics (Ashraf et al., 2013), organic acids (Elbaz et al., 2021), herbal extracts (Sugiharto, 2020), and guanidinoacetic acid (Majdeddin et al., 2020), among others, have shown to attenuate the negative effects of HS. It was reported that a diet with 1.2 g/kg guanidinoacetic acid could improve feed efficiency and survival during chronic cyclic HS, which was associated with enhanced breast muscle energy status and arginine sparing effect (Majdeddin et al., 2020). Analogously, dietary 0.069 mg/kg 25-OH-D_3_ supplementation in broilers was noticed to restore the decrease in body weight (**BW**), weight gain, and FI induced by chronic cyclic HS, along with improved intestinal barrier (Zhang et al., 2021).

Methyl sulfonyl methane (**MSM**) also known as dimethyl sulfone with the formula (CH_3_)_2_SO_2_ is a stable metabolite of dimethyl sulfoxide and is involved in the sulfur cycle in nature. In human clinical practice, MSM has benefits that may extend to a plethora of diseases such as obesity and arthritis and pain due to joint degeneration for which it has been extensively used as a dietary supplement to improve human health (Usha and Naidu, 2004; Butawan et al., 2017). There is evidence that MSM has diverse functions such as anti-inflammatory, anticancer, antioxidant, antiallergy, and anti-immunosuppressive activities (Butawan et al., 2017). It is paramount to understand how MSM can exert its bioactivities in the animal's body. First, sulfur from MSM might be used by gut microbiota to synthesize the sulfur amino acids methionine and cysteine, and consequently these amino acids can be absorbed by the intestine and incorporated in animal tissues (Butawan et al., 2017). However, it can be assumed that this contribution to the circulatory sulfur amino acid pool may not be quantitatively meaningful in monogastric meat producing animals. Second, MSM as such can be also absorbed by the small intestine in a passive and carrier-independent manner with high capacity (Wong et al., 2017), subsequently be converted to sulfate in the body and be used for post-translational sulfation of proteins and other sulfate reactions such as sulfation of mucins and cartilage proteoglycans. This latter process involves the transfer of sulfate from phosphoadenosine-5’-phosphosulfate (**PAPS**) to various substrates by sulfotransferases (Miller and Schmidt, 2020). In the animal's metabolism, commonly the primary source of sulfate used in this pathway is the oxidation of cysteine. In this respect, MSM could spare cysteine that then can be used for other purposes such as the synthesis of the endogenous antioxidant glutathione (**GSH**) and taurine (Wong et al., 2017). Dietary sulfate may also contribute to the body pool of sulfate, yet its intestinal absorption is mediated by the sodium cotransporter NaSi-1 and possibly others and might be rate limiting for adequate intake (Beck and Silve, 2001). Third, the role of MSM as methyl donor has been debated (Butawan et al., 2017). Finally and most importantly, a part of being a potential source of sulfur amino acids and sulfate MSM as such is bioactive and believed to operate at the crosstalk of inflammation and oxidative stress at the transcriptional and subcellular level (Butawan et al., 2017). Its anti-inflammatory mechanism stems from the downregulation of the transcriptional activity of nuclear factor kappa-light-chain-enhancer of activated B cells (**NF-κB**), resulting in lower expression of proinflammatory cytokines, inducible nitric oxide synthase and cyclooxygenase-2. As these mediators may result in elevated ROS production (e.g., superoxide radical and nitric oxide), MSM indirectly reduces oxidative stress. Analogously, MSM has shown to repress the expression and activities of signal transducers and activators of transcription (**STAT**), which regulates genes involved in apoptosis, differentiation, and proliferation, all of which generate ROS as a necessary signaling component. Moreover, MSM fosters the nuclear factor (erythroid-derived 2)-like 2 (**Nrf2**) translocation to the nucleus. Nrf2 is well documented for its association with antioxidant enzymes like SOD, glutathione peroxidase (**GPx**) and glutamate-cysteine ligase (**GCL**), the latter catalyzing the rate-limiting step in GSH synthesis. Regarding broilers, orally administered MSM was easily detectable in plasma and widely distributed in tissues of the body and could cross the blood-brain barrier (Abdul Rasheed et al., 2019). It means that it is very plausible dietary MSM may exert its bioactivities in broilers. Besides, MSM at either acute (single dose at 1,000–2,000 mg/kg BW) or subchronic (1,500 g/kg BW daily for 21 d) concentrations did not cause any adverse effects on growth or clinical outcomes, underscoring its low toxicity. In layers, MSM deposition in the egg albumen linearly increased in groups fed up to 4.0 g/kg MSM (Kim et al., 2022).

Hence, in recent years, the effects of MSM are increasingly being investigated in meat producing animals, not in the least in poultry. In pigs, dietary 0.6 g/kg MSM increased average daily FI (**ADFI**) in grower-finishers (Jang et al., 2006), whereas in the study of Cho et al. (2005) dietary MSM (0.1 and 0.2 g/kg) resulted in equivocal effects on nutrient digestibility and feed efficiency. More evidence is available in meat-type domestic birds (Supplementary Table 1). In summary, MSM improved performance and lowered mortality (Liu and Zhou, 2008; Hwang et al., 2017; Jiao et al., 2017; Yan et al., 2020), enhanced antioxidant defenses and reduced markers of oxidation (Hwang et al., 2017; Rasheed et al., 2020a; Yan et al., 2020; Miao et al., 2022), and showed immunosuppressive properties (Yan et al., 2020; Miao et al., 2022). Finally, breast quality of MSM-fed poultry exhibited higher water-holding capacity (**WHC**) and lower drip loss, higher redness (a*), and lower concentrations of TBARS (Hwang et al., 2017; Jiao et al., 2017; Yan et al., 2020). It must be noted that in these studies (Supplementary Table 1) diverging dosages of dietary MSM were applied, for example, Liu and Zhou (2008) fed 0.250 g/kg MSM to meat ducks while Rasheed et al. (2020b) applied 4 g/kg MSM in Ross 308 broilers, the latter in a *Eimeria* challenge model. Altogether, it remains appealing to evaluate the potential of MSM to attenuate the effects of HS, a well-established condition to inflict oxidative stress (Akbarian et al., 2016) and inflammation (Zhang et al., 2021). We therefore hypothesized that dietary MSM supplementation could attenuate HS-induced detrimental effects in finishing broilers mediated by enhancement of antioxidant defenses. The present study tested 1 and 2 g/kg MSM supplied during the entire rearing of Ross 308 broilers that were subjected to chronic cyclic HS in the finisher phase.

## MATERIALS AND METHODS

The study was conducted in accordance with the ethical standards and recommendations for accommodation and care of laboratory animals covered by the European Directive (2010/63/EU) on the protection of animals used for scientific purposes and by the Belgian royal decree (KB29.05.13) on the use of animals for experimental studies. All procedures and animal handlings were approved by Ethics Committee of Faculty of Veterinary Medicine, Ghent University (No. EC2018-71).

### Birds and Husbandry

Broilers were housed in a single climate-controlled room which contained 36 pens. Each pen had a dimension of 0.8 m × 1.7 m and had concrete floor covered with wood shavings. Pens were equipped with 2 nipples for drinking water (tap water) per pen (Swii'Flo, Roxell; Maldegem, Belgium) and 1 feeder pan (MiniMax, Roxell). The experiment had 3 rearing phases: starter (from d 0 to 10), grower (from d 10 to 21), and finisher (from d 21 to 39). The light schedule was 23L:1D and 18L:6D (18 L from 04:00 to 22:00) during 1 to 7 d and beyond, respectively. Temperature in starter and grower phase was set to decrease linearly from 34°C on d 0 until a basal temperature of 22°C on d 24. The chronic cyclic HS model was implemented from d 24 to 39 as shown in Supplementary Figure 1. The temperature was increased from 22 to 34°C for 6 h/d (from 9:00 to 15:00) with relative air humidity (**RH**) between 52 and 58%, however on some days exceeding 58% in this 6 h interval (Supplementary Figure 1). From 8:00 to 9:00, the temperature was increasing from 22 to 34°C, and from 15:00 to 16:00, the temperature went down from 34 to 22°C. The rest of the day the temperature was 22°C. The room was equipped with heaters with thermostats and ventilators to enable the desired temperature. RH was adjusted with an automated water nebulization system. The temperature and the RH were recorded at 10 min intervals with a dry bulb thermo-hygrometer (Escort RH iLog, Escort Verification Technologies Inc., Buchanan, United States). The chickens were fed ad libitum and had free access to water during the whole experiment.

### Experimental Design

A total of seven hundred ninety-two 1-day-old male Ross 308 broilers (43.3 ± 0.31 g; Vervaeke-Belavi hatchery, Tielt, Belgium) were randomly assigned to 3 groups with 12 replicate pens of 22 birds each in a completely randomized block design. Dietary treatments were applied in all rearing phases and were as follows: 1) Ctrl, basal diet; 2) 1 g/ kg MSM, basal diet + 1 g/kg MSM on-top; and 3) 2 g/kg MSM, basal diet + 2 g/kg MSM on-top. Wheat and soybean-based feeds were formulated to meet the nutrient requirements of broilers, according to Ross 308 recommendations. To fine-tune the formulation, all major ingredients (wheat, soybean meal, and toasted soybeans) were analyzed for nutrient composition by near infrared spectroscopy by Evonik Operations GmbH (Hanau-Wolfgang, Germany). Ingredient and nutrient composition of basal diets are given in Table 1, while analyzed composition is given in Supplementary Table 2. All the diets were provided as pellets during all rearing phases.

**Table 1:** Composition of wheat-soybean based basal diets (as-fed).

Item	Starter (d 0–10)	Grower (d 10–21)	Finisher (d 21–39)
Ingredients, %			
Wheat	59.8	67.3	69.0
Soybean meal (CP48%)	23.5	15.8	13.1
Toasted full-fat soybeans	10.0	10.0	10.0
Lard	1.00	2.00	3.00
Soybean oil	0.818	0.443	0.731
Monocalcium phosphate	1.26	1.10	0.966
Limestone	1.66	1.54	1.46
Salt	0.232	0.181	0.203
Sodium bicarbonate	0.250	0.280	0.251
Premix vitamins and minerals^1^	0.500	0.500	0.500
Choline chloride (50%)	0.100	0.100	0.050
6-Phytase (500 FTU)	0.010	0.010	0.010
L-Lys	0.329	0.308	0.313
DL-Met	0.333	0.24	0.218
L-Thr	0.165	0.131	0.127
L-Val	0.088	0.049	0.046
Xylanase + glucanase	0.010	0.010	0.010
Formulated nutrient content, %			
Dry matter	89.4	89.4	89.0
Metabolizable energy, kcal/kg	3000	3100	3200
Crude protein	23.1	20.3	19.2
Ether extract	5.81	6.39	7.69
Crude ash	6.32	5.68	5.35
Starch	35.8	40.3	41.2
Calcium	0.96	0.87	0.81
Available phosphorus	0.48	0.44	0.41
Na+K-Cl, meq/100 g	25.0	22.0	20.5
Lys	1.38	1.17	1.10
Dig. Lys	1.24	1.05	0.99
Dig. Met+Cys/dig. Lys	75	75	75
Dig. Thr/dig. Lys	67	67	67
Dig. Trp/dig. Lys	21	21	21
Dig. Val/dig. Lys	80	80	80
Dig. Arg/dig. Lys	106	105	104
Dig. Iso/dig. Lys	67	68	67
Dig. Leu/dig. Lys	116	119	119

Abbreviations: Arg, arginine; Cl, chlorine; Cys, cysteine; Dig., digestible; Dig. Trp, tryptophan; Iso, isoleucine; K, potassium; Leu, leucine; Lys, lysine; Met, methionine; Na, sodium; Thr, threonine; Val, valine.

1 Premix providing per kg of diet: vitamin A (retinyl acetate), 10,000 IU; vitamin D_3_ (cholecalciferol), 2,500 IU; vitamin E (dl-α-tocopherol acetate), 50 mg; vitamin K_3_ (menadione), 1.5 mg; vitamin B_1_ (thiamine), 2.0 mg; vitamin B_2_ (riboflavin), 7.5 mg; niacin, 35 mg; D-pantothenic acid, 12 mg; vitamin B_6_ (pyridoxine-HCl), 3.5 mg; vitamin B_12_ (cyanocobalamine), 20 µg; folic acid, 1.0 mg; biotin, 0.2 mg; choline chloride, 460 mg; Fe (FeSO_4_·H_2_O), 80 mg; Cu (CuSO_4_·5H_2_O), 12 mg; Zn (ZnO), 60 mg; Mn (MnO), 85; I (Ca(IO_3_)_2_), 0.8 mg; Co (Co_2_CO_3_(OH)_2_), 0.77 mg; Se (Na_2_O_3_Se), 0.15 mg.

On d 23, 25, and 39, 1 bird per pen with a weight close to the average weight of the pen was selected. Sampling on d 25 and 39 started minimum 3 h after inducing HS on that day. Birds were taken out of the pen carefully and rectal temperature was determined. Next, an anesthetic combination (2 mg/kg BW xylazine and 10 mg/kg BW ketamine) was injected in the left breast muscle (middle of length) using a 22 G needle. The anesthetized bird was placed in an individual dark box in the experimental room (maintaining temperature and RH conditions) until euthanasia. After 10 min and control of complete anesthesia, euthanasia was induced by a lethal dose (60 mg/kg BW) of pentobarbiturate. Then, blood was taken by puncture in heart with an 80 mm needle 22 G and collected in various tubes. The K_2_EDTA-tube was used for harvesting plasma, followed by storage at −80°C, pending analysis of heat shock protein 70 (**HSP70**), TBARS, and GPx and SOD activity. Serum was stored at −20°C and used for triiodothyronine (**T3**), thyroxine (**T4**), and nitric oxide (**NO**) quantification. Erythrocytes were harvested by centrifuging (3,000 × *g*, 15 min) 0.5 mL of heparinized blood with bathophenanthrolinedisulfonic acid disodium salt added as metal chelator and removing the residual plasma (Degroote et al., 2020). Erythrocytes were lyzed with 70% metaphosphoric acid solution and intense vortexing. An aliquot of this acid extract was transferred to a vial, containing γ-glutamyl-glutamate as an internal standard, and was snap frozen in liquid nitrogen before storing at −80°C prior to quantitation of GSH and glutathione disulfide (**GSSG**). Immediately after euthanasia, liver (2 × 2 cm^2^ middle part of right liver lobe) and breast muscle (2 × 2 × 2 cm^3^ piece of the middle part of right pectoralis major) were sampled. Samples were snap frozen in liquid nitrogen, stored at −80°C pending determination of TBARS, GPx and SOD activity, GSH, and GSSG.

### Growth Performances

Pen live BW and feed leftovers were recorded at d 0, 10, 21, and 39. Average daily gain (**ADG**), ADFI, feed-to-gain ratio (**F:G**, adjusted for mortality and calculated as total feed intake divided by total gain including the weight of lost birds per pen), and mortality rate (**MR**) were calculated for each rearing period. For the total period, a corrected feed-to-gain ratio, that is, F:G_corrected_, was calculated by correcting to a similar final BW of 3,050 g for all pens by assuming 1.76 points increase per 100 g BW (based on Male Ross308 Performance Objectives 2019) using the formula:

$$F:G_{\text{corrected}}=\;F:G+\frac{\left(BW_{d39}-3.050\right)}{10}\times \;1.76$$

where BW_d39_ is the average BW at d 39 (kg). Of note, for each day in the chronic cyclic HS model, FI for period of 6 h of HS and for rest of the day was measured, and hence the percentage of FI for period of 6 h of HS to the total FI consumed was calculated daily (**FI_HS_**). In addition, the European Production Efficiency Factor (**EPEF**) was calculated:

$$\text{EPEF}=\;\frac{\left(100-\text{MR}\right)\times BW_{d39}}{39\;\times \;F:G}\times \;100$$

where MR is the mortality rate for the total period (%), BW_d39_ is the average BW at d 39 (kg), and F:G is the feed-to-gain ratio (g/g) for the total period.

### Panting Frequency and Rectal Temperature

On d 24, 31, and 38 after 4 h of HS, 2 chickens per pen were selected *at random* and rectal temperature was determined immediately using a digital thermometer inserted to a minimum depth of 3 cm in the cloaca. Panting frequency defined as the number of breaths per minute was measured on the same occasion. In detail, videos were recorded for at least 1.5 min for each pen, then 3 birds were randomly selected for assessment of the panting frequency during a 1 min interval.

### Carcass Yield and Portions

At 40 d of age (no HS that day), following 12 h fasting, 1 bird per pen with weight close to average weight of the pen was selected. After euthanasia, they were bled and defeathered. The carcass weight including skin and without internal organs, feet, head, neck, and abdominal fat was measured. The abdominal fat was weighed. Breast (pectoralis major and minor) was removed from the carcass, trimmed of skin and bone, and weighed. Thigh and drumstick with skin and bone were weighed. Carcass yield, the percentages of abdominal fat, breast, thigh, and drumstick, were then expressed as a percentage of live body weight.

### Breast Meat Quality

Also on 40 d, 1 bird per pen with weight close to average weight of the pen was euthanized. Breast muscle (pectoralis major and minor) were then used for meat quality assessment upon slaughter (0 h) and 24 h postmortem as described by Michiels et al. (2014). The eviscerated carcasses were transferred to the chilling room (4°C) immediately after slaughter. pH of breast muscle was measured using a pH meter. Light (L*), redness (a*), and yellowness (b*) values were measured on breast muscle samples with a HunterLab Miniscan XE plus spectrocolorimeter (light source D65, standard observer 10°, 45°/0° geometry, 1-inch light surface, white standard). Drip loss was evaluated through the proportionate weight loss of a sample hanging in a plastic bag for 24 and 72 h at 4°C. Press loss was determined based on the volume of free water squeezed from ground muscle sample using a filter paper press method. The breast muscle was used for assessment of myopathies in their pectoralis major (wooden breast and white striations). The incidence of myopathies was measured at 0 and 24 h postmortem by using a severity scale, ranging from 0 to 3, where 0 refers to breast fillets considered normal, 1 indicates moderate myopathy, 2 indicates severe myopathy, and 3 refers to extreme myopathy, adapted from Kuttapan et al. (2016). Regarding oxidative stability, defined subsamples of the pectoralis major that had been frozen and thawed were wrapped in oxygen permeable polyethylene film and displayed under fluorescent light (1,000 lux) for 5 d at 4°C to simulate retail display. Lipid oxidation was assessed by measuring TBARS using the distillation method and was expressed as μg of MDA per gram of meat.

### Plasma HSP70 and Thyroid Hormones

HSP70 in plasma was determined employing the competitive inhibition enzyme immunoassay technique (Chicken HSP70, Code CSB-E11196Ch, Cusabio, Wuhan, China) following kit recommendations. The hormones T3 and T4 in serum were determined by competitive radioimmunoassay (Zoolyx; Aalst, Belgium). Serum NO was assessed via the nitrate/nitrite colorimetric assay kit (Item No. 780001; Cayman Chemical, Ann Arbor, MI) which measures the sum of nitrate and nitrite, the final products of NO reactions in the body (Chapman and Wideman, 2006).

### Oxidative Stability Measures for Blood, Liver, and Breast Muscle

The buffered aqueous extracts of liver and breast were prepared after mixing with 1% Triton X-100 phosphate buffer (pH 7; 50 mmol/L), and then homogenized, centrifuged, and filtrated. Subsequently, the TBARS method was used to assess lipid oxidation in buffered aqueous extracts and plasma with MDA as standard using spectrophotometry at 532 nm according to Grotto et al. (2007). GPx activity were quantified based on the dynamical alteration in the oxidation of NADPH and reaction time using Multi-Mode Microplate Readers at 340 nm (Hernández et al., 2004). This was determined in of buffered aqueous extracts and plasma, and activity was defined as the amount of sample (g) or l mL plasma required to oxidize 1 μmol of 2,4-dinitrophenylhydrazine (**DNPH**) per minute at 25°C. The SOD activity was determined by observing the increase in absorbance at 420 nm for 5 min by spectrophotometry (Marklund and Marklund, 1974). One unit of SOD activity was defined as the amount of extract required to inhibit the rate of NADH oxidation by the control (no SOD) by 50%. Liver and breast muscle samples were homogenized in a 10% perchloric acid solution. After centrifugation (13,000 × *g*, 15 min, 4°C), the resulting acid extract was transferred to a tube containing a γ-glutamyl-glutamate internal standard solution. Next, GSH and GSSG in acid extracts were quantitated by high-performance liquid chromatography (**HPLC**) analysis (Degroote et al., 2020). Iodoacetic acid was used as a thiol quenching agent, 1-chloro-2,4-dinitrobenzene as derivatization reagent, reversed-phase HPLC separation was done on an aminopropyl column with absorption measurement at 365 nm. The GSH and GSSG concentrations were determined relative to internal and external standard solutions and were expressed on fresh samples basis.

### Statistical Analysis

The data obtained were analyzed by the Kolmogorov-Smirnov and Levene's tests to assess normal distribution and homogeneity of variances, respectively, using SAS 9.0 (SAS Institute Inc., Cary, NC). Data for the mortality were evaluated using the nonparametric Kruskal-Wallis test. For remainder variables, normal distribution and homogeneity of variances were confirmed. The comparisons were performed with one-way analysis of variance (**ANOVA**) with Tukey's multiple comparison post hoc test using the following statistical model:

$$Y_{j}=\mu +D_{j}+\varepsilon_{j}$$

Thereby is *Y_j_* the mean value of treatment *j* (Ctrl, 1 g/kg MSM, and 2 g/kg MSM), *μ* is the overall mean, *Dj* is the fixed effect of treatment *j*, and $\varepsilon_{j}$ is the error term. Block was further included as random factor if significant. In case, day (e.g., FI_HS_, rectal temperature) was included as within-subject factor (repeated measure). As time after initiating HS may affect the birds’ metabolic response, time after starting HS for physiological variables assessed on sampled broilers on d 24, 31, and 38 was included as covariate if significant. Orthogonal contrasts were applied to test for linear and quadratic effects upon incremental levels of MSM supplementation in the diet. For all analyses, pen was considered the experimental unit. Means are given as least square means. Furthermore, referring to mortality, survival curves were constructed using the Kaplan-Meier representation and statistically analyzed using the log-rank test (GraphPad Prism 5.00; San Diego, CA). For wooden breast and white striations statistical evaluation was done by the chi-square test (SAS 9.0). Differences were considered significant when *P* < 0.05 and as a trend when *P* < 0.1.

## RESULTS

### Growth Performances

**Figure 1:**
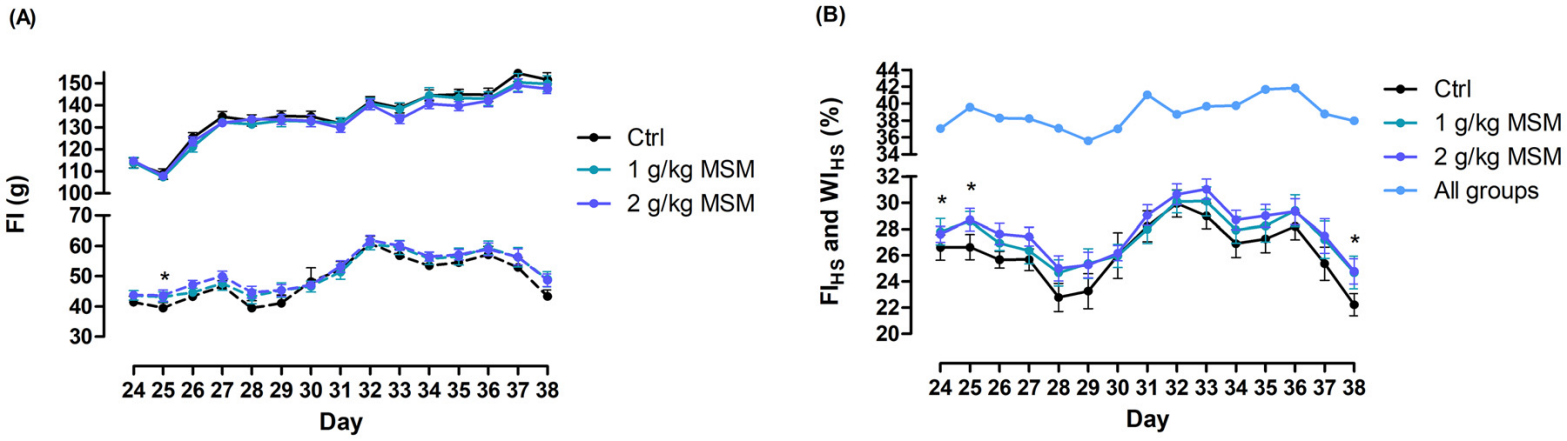
(A) Feed intake (FI) per bird and per day during period of 6 h of heat stress (broken lines) and during rest of the day (full lines) in period d 24 till 38. (B) Percentage of feed intake (FI_HS_) or water intake (WI, for all groups combined) that was consumed during 6 h of heat stress (HS) to total intake in period d 24 till 38. * Denotes that there was a trend for linear effect (*P* < 0.1) of incremental levels of MSM in the diet. MSM = methyl sulfonyl methane. Broilers were fed a wheat-soybean starter diet from d 0 to 10, a grower diet from d 10 to 21 and a finisher diet from d 21 to 39. The chronic cyclic heat stress model was implemented from d 24 till 39. On d 28 to 31 increased mortality was noticed; in order to prevent excessive mortality, the high temperature during 6 h/d was decreased to 32°C in period d 32 to 37 and to 33°C in period d 38 to 39; leading to higher FI_HS_.

**Figure 2:**
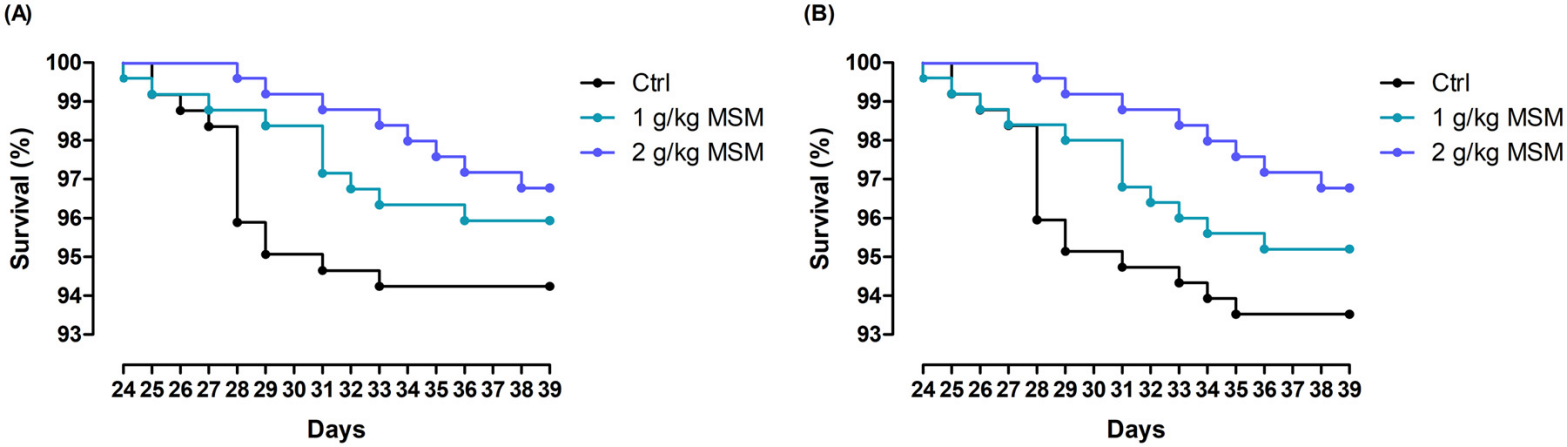
(A) Percent survival of birds in period of chronic cyclic heat stress model (d 24–39) using Kaplan-Meier representation based on mortality. Log-rank test, *P* = 0.159. (B) Percent survival of birds in period of chronic cyclic heat stress model (d 24–39) using Kaplan-Meier representation based on mortality and culling. Log-rank test, *P* = 0.087. Abbreviation: MSM, methyl sulfonyl methane. Broilers were fed a wheat-soybean starter diet from d 0 to 10, a grower diet from d 10 to 21 and a finisher diet from d 21 to 39. The chronic cyclic heat stress model was implemented from d 24 till 39.

Birds were found healthy throughout the study and performed altogether very well, with final BW exceeding 3 kg. In the starter period, no significant effects were found for either performance indicator (Table 2). In grower period, the experimental treatments did not result in differences in terms of ADFI. In contrast, BW and ADG showed linear increases with higher inclusion of MSM in the diet (*P* < 0.05). ADG in this period was 2.6 and 3.4% higher for 1 and 2 g/kg MSM, respectively, as compared to Ctrl (both *P* < 0.05). F:G was reduced by 6 and 7 points for 1 and 2 g/kg MSM when compared to Ctrl, respectively (both *P* < 0.05). In the starter + grower period, MSM linearly improved ADG due to better feed efficiency (-3 points are compared to Ctrl) (both *P* < 0.05), whereas ADFI decreased in a linear manner (*P* < 0.05). The finisher period encompassed 2 subperiods: d 21 to 24 was without HS protocol, whereas in period d 24 to 39 the HS protocol prevailed, reducing potential growth of birds. None of the performance indicators was affected by treatment. Neither for the total period, performance was affected by diet. Figure 1A illustrates a steady increase of absolute FI during time of lower temperatures over the 14-day period, while it was stable for absolute FI during 6 h of high temperatures unless T was decreased to 32°C in period d 32 to 37. Although FI_HS_ for period d 24 to 38 did not differ among treatments, FI_HS_ for d 24, 25, and 38 showed a trend for linear effect by incremental levels of MSM (Figure 1B). Relative water intake (**WI_HS_**) during time of high temperatures (consistently above 36%) was substantially higher than relative feed intake, the latter showing considerable fluctuations between 22 and 30% (Figure 1B). According to Table 2, mortality in finisher period accounted for 6.3, 4.7, and 3.1% in Ctrl, 1 g/kg MSM, and 2 g/kg MSM in the finisher period, respectively. Yet, mortality in Ctrl occurred mostly in the beginning of the chronic cyclic HS model, with peak mortality on d 28, whereas in the 2 g/kg MSM group dead birds were found mostly in the second half of this period (Figure 2A). A trend suggests differences in survival curves across the treatments when mortality and culling was included (Figure 2B, *P* = 0.087).

**Table 2:** Dietary effect on body weight (BW), average daily gain (ADG), average daily feed intake (ADFI), the feed-to-gain ratio (F:G), mortality, and European production efficiency factor (EPEF) in male broilers subjected to chronic cyclic heat stress.

Item	Ctrl	1 g/kg MSM	2 g/kg MSM		*P-*value		
				SEM	Model	Linear	Quadratic
Starter (d 0–10)							
Initial BW, g	41.3	41.4	41.2	0.05	0.176	0.289	0.123
Final BW, g	362	359	357	1.2	0.258	0.105	0.834
ADG, g/d	32.0	31.7	31.6	0.12	0.266	0.111	0.783
ADFI, g/d	32.5	32.5	32.3	0.12	0.665	0.444	0.638
F:G	1.02	1.03	1.03	0.001	0.150	0.057	0.716
Mortality, %	1.1	0.0	0.8		0.357		
Grower (d 10–21)							
Final BW, g	1171	1189	1194	4.3	0.066	0.027	0.453
ADG, g/d	73.6^b^	75.5^a^	76.1^a^	0.35	0.004	0.002	0.320
ADFI, g/d	105	103	102	0.5	0.171	0.085	0.454
F:G	1.42^a^	1.36^b^	1.35^b^	0.009	<0.001	<0.001	0.104
Mortality, %	0.0	1.1	0.4		0.147		
Starter + grower (d 0–21)							
ADG, g/d	53.8	54.7	54.9	0.20	0.065	0.026	0.463
ADFI, g/d	70.1	68.8	68.8	0.25	0.054	0.035	0.220
F:G	1.20^a^	1.17^b^	1.17^b^	0.005	0.003	0.003	0.079
Mortality, %	1.1	1.1	1.1		0.908		
Finisher (d 21–39)							
Final BW, g	3081	3100	3066	11.5	0.491	0.566	0.326
ADG, g/d	106	106	104	0.6	0.212	0.174	0.333
ADFI, g/d	177	179	178	1.0	0.775	0.731	0.609
FI_HS_^1^, %	26.2	27.9	27.9	0.43	0.134	0.129	0.368
F:G	1.69	1.71	1.72	0.008	0.269	0.108	0.907
Mortality, %	6.3	4.7	3.1		0.399		
Total (d 0–39)							
ADG, g/d	77.9	78.4	77.6	0.30	0.495	0.569	0.329
ADFI, g/d	116	116	117	0.6	0.842	0.627	0.918
F:G	1.54	1.53	1.54	0.004	0.891	0.912	0.641
F:G corrected^2^	1.54	1.54	1.54	0.004	0.802	0.523	0.879
Mortality, %	7.4	5.9	4.2		0.472		
EPEF^3^	476	488	491	6.4	0.561	0.362	0.761

Broilers were fed a wheat-soybean starter diet from d 0 to 10, a grower diet from d 10 to 21, and a finisher diet from d 21 to 39. The chronic cyclic heat stress model was implemented from d 24 till 39.

a,b Values with different superscripts within a row are significantly different at *P* < 0.05 (*n* = 12). Abbreviation: MSM, methyl sulfonyl methane.

1 Indicates the percentage of total feed intake that was consumed during hours of heat stress (6 h/d) in period d 24 to d 38. Statistics was done including day as within subject factor (repeated measure).

2 F:G for total period corrected to final body weight of 3,050 g by assuming 1.76 points increase per 100 g body weight (based on Male Ross308 Performance Objectives 2019).

3 $\text{EPEF}=\;\frac{\left(100-\text{MR}\right)\times BW_{d-39}}{39\;\times \;F:G}\times \;100.$

### Rectal Temperature and Panting Frequency

The supplementation with MSM resulted in numerically lower rectal temperatures at all days (Table 3). On d 38, the diets with graded levels of MSM resulted in a linear effect on rectal temperature (*P* < 0.05). In total, rectal temperature was significantly reduced by 2 g/kg MSM in the diet as compared to Ctrl (−0.3°C, *P* < 0.05). Panting occurred heavily during episodes of HS, and apparently increased when birds’ age, although d 38 was comparable to d 31 likely because of lower stable temperatures on d 38 as compared to d 31. At d 24, a significant treatment effect was found which indicates that panting frequency was reduced by 2 g/kg MSM as compared to Ctrl, and the effect at d 24 was linear (*P* < 0.05).

**Table 3:** Dietary effect on rectal temperature and panting frequency in male broilers subjected to chronic cyclic heat stress.

Item	Ctrl	1 g/kg MSM	2 g/kg MSM	SEM	*P-*value		
					Model	Linear	Quadratic
Rectal temperature, °C							
d 24	42.5	42.3	42.3	0.06	0.304	0.154	0.540
d 31	43.0	42.9	42.6	0.09	0.252	0.106	0.761
d 38	42.9	42.7	42.6	0.07	0.118	0.040	0.990
Total^1^	42.8^a^	42.6^ab^	42.5^b^	0.03	0.013		
Panting frequency, #/min							
d 24	134^a^	119^ab^	112^b^	4.1	0.035	0.012	0.508
d 31	154	158	143	4.4	0.347	0.315	0.292
d 38	153	147	152	2.9	0.638	0.894	0.350
Total^1^	148	141	137	2.8	0.200		

Broilers were fed a wheat-soybean starter diet from d 0 to 10, a grower diet from d 10 to 21 and a finisher diet from d 21 to 39. The chronic cyclic heat stress model was implemented from d 24 till 39.

a,b Values with different superscripts within a row are significantly different at *P* < 0.05 (*n* = 12). Abbreviation: MSM, methyl sulfonyl methane.

1 For all days, with statistics done including day as within subject factor (repeated measure).

### Carcass Yield and Portions and Breast Meat Quality

No significant effects on carcass yield and portions were found (Table 4), though trends for quadratic effects suggest lowest carcass yield (*P* = 0.073) due to lower breast meat yield (*P* = 0.069) by 1 g/kg MSM. Regarding to the characteristics of breast meat (Table 5), pH at slaughter and 24 h postmortem were not changed by the diet, with ultimate values 0.8 lower than initial pH. As far as color is concerned, a consistent reducing effect of MSM supplementation on L* and b* might be conceived, though only significance was seen for b* 24 h postmortem with 1 g/kg MSM which was lower than Ctrl (*P* < 0.05). Hence a quadratic effect on b* 24 h was found (*P* < 0.05). Drip and press loss were not affected by diet. A significant effect on white striations 24 h postmortem was observed. The results suggest that more cases with moderate white striations occurred when MSM was supplemented at 2 g/kg MSM as compared to other treatments. However, no cases of severe white striations were seen. Finally, TBARS after simulated retail display of breast meat showed a linear trend for lower levels of the standard MDA upon increased inclusion of MSM (*P* = 0.078).

**Table 4:** Dietary effect on carcass yield and portions (%) in male broilers subjected to chronic cyclic heat stress after overnight fasting on d 40.

Item	Ctrl	1 g/kg MSM	2 g/kg MSM	SEM	*P-*value		
					Model	Linear	Quadratic
Abdominal fat	1.1	1.1	1.1	0.05	0.966	0.878	0.838
Carcass yield	75.9	74.9	75.9	0.26	0.196	0.983	0.073
Breast	24.2	23.4	24.1	0.19	0.182	0.784	0.069
Thigh	11.2	11.1	11.2	0.11	0.999	0.986	0.966
Drumstick	9.9	10.0	10.2	0.07	0.311	0.129	0.961

Broilers were fed a wheat-soybean starter diet from d 0 to 10, a grower diet from d 10 to 21, and a finisher diet from d 21 to 39. The chronic cyclic heat stress model was implemented from d 24 till 39. Values with different superscripts within a row are significantly different at *P* < 0.05 (*n* = 12).

Abbreviation: MSM, methyl sulfonyl methane.

**Table 5:** Dietary effect on breast meat quality in male broilers subjected to chronic cyclic heat stress overnight fasting on d 40.

Item	Ctrl	1 g/kg MSM	2 g/kg MSM	SEM	*P-*value		
					Model	Linear	Quadratic
pH							
0 h	6.8	6.8	6.8	0.03	0.853	0.796	0.615
24 h	6.0	6.0	6.0	0.02	0.385	0.517	0.229
Color L*							
L* 0 h	57.9	56.7	56.9	0.51	0.613	0.425	0.550
a* 0 h	3.2	3.6	3.5	0.19	0.759	0.539	0.672
b* 0 h	15.9	15.4	15.5	0.26	0.734	0.541	0.616
L* 24 h	57.8	56.7	56.2	0.59	0.549	0.283	0.849
a* 24 h	5.0	5.0	5.3	0.16	0.751	0.556	0.650
b* 24 h	18.1^a^	16.7^b^	17.3^ab^	0.22	0.026	0.116	0.022
Drip loss, %							
0–24 h	2.3	2.0	1.8	0.19	0.615	0.329	0.921
0–72 h	3.1	2.7	2.7	0.21	0.673	0.407	0.728
Press loss, %	20.3	20.4	20.4	0.31	0.987	0.875	0.994
Wooden breast							
0 h^1^	21/1/0	19/3/0	22/2/0		0.565		
24 h^1^	14/8/0	15/7/0	12/12/0		0.420		
White striations	19/3/0	21/1/0	20/4/0		0.420		
0 h^1^	19/3/0	21/1/0	20/4/0		0.420		
24 h^1^	21/1/0	21/1/0	18/6/0		0.044		
TBARS, µg MDA/g	0.157	0.125	0.122	0.0083	0.155	0.078	0.406

Broilers were fed a wheat-soybean starter diet from d 0 to 10, a grower diet from d 10 to 21 and a finisher diet from d 21 to 39. The chronic cyclic heat stress model was implemented from d 24 till 39.

a,b Values with different superscripts within a row are significantly different at *P* < 0.05 (*n* =12). Abbreviations: MDA, malondialdehyde; MSM, methyl sulfonyl methane; TBARS, thiobarbituric acid reactive substances.

1 Wooden breast and white striations: normal, 0; moderate, 1; and severe, 2. Counts per level are given in order of severity and statistical evaluation was done by the chi-square test (*n* = 22–24).

### Physiological Measurements in Blood

On d 23, 25 and 39, 1 bird per pen with a weight close to the average weight of the pen was selected. Sampling on d 25 and 39 started minimum 3 h after inducing HS on that day. BW and rectal temperature of sampled birds are summarized in Supplementary Table 3. Rectal temperature of sampled birds on d 23 represents values for thermoneutral conditions, and thus ranged between 41.1 and 41.3°C without treatment differences. However, on d 25 rectal temperature was largely elevated and obviously affected by diet (*P* < 0.05). Rectal temperature linearly decreased with higher level of MSM in the diet, both supplemented groups being different from Ctrl (*P* < 0.05), and for 2 g/kg MSM this was 0.5°C lower than Ctrl. This rectal temperature is slightly higher than those on d 24 as shown in Table 3, at least for Ctrl and 1 g/kg MSM, suggesting increased disturbance of body temperature homeostasis in these groups on the second day of HS.

No treatments effects on HSP70 could be perceived on either sampling day, apart from a trend for linear reduction on d 23, that is, before the start of the chronic cyclic HS model (*P* = 0.084) (Table 6). It also appears that plasma HSP70 showed slight decreases upon acute HS (d 25), whereas after 2 wk of HS (d 39) levels rose substantially with large variation across samples. T4 could not be detected on d 23, while higher values were found on d 25 and 39. A trend for linear increase on d 39 by higher MSM dosage was observed (*P* = 0.091). T3, the derivative of T4, and most active form, was reduced on d 23 by feeding 2 g/kg MSM as compared to Ctrl (*P* < 0.05). On d 39, a trend suggests that T3 was linearly elevated by MSM (*P* = 0.059). Irrespective of treatment, a sharp reduction in circulating T3 levels as birds age and thus subjected for longer period to HS is obvious. The ratio T3/T4 reflecting the conversion efficiency from T4 to T3 was not altered by diet.

**Table 6:** Dietary effect on parameters in blood in male broilers subjected to chronic cyclic heat stress.

Item	Matrix	Ctrl	1 g/kg MSM	2 g/kg MSM	SEM	*P-*value		
						Model	Linear	Quadratic
HSP70, ng/mL	Plasma							
d 23		1.69	1.69	1	0.164	0.139	0.084	0.323
d 25								
d 39								
T4, μg/dL	Serum							
d 23^1^		1.09	1.07	1.08	0.077	0.995	0.983	0.926
d 25		3.63	2.77	4.23	0.438	0.406	0.58	0.223
d 39		0.5	0.53	0.5	0.019	0.771	0.984	0.475
T3, μg/L	Serum							
d 23		0.54	0.6	0.63	0.022	0.227	0.091	0.793
d 25^2^		2.50^a^	2.43^a^	2.06^b^	0.065	0.009	0.005	0.215
d 39		1.5	1.74	1.72	0.064	0.199	0.139	0.306
T3/T4	Serum							
d 23^1^		0.97	1.1	1.12	0.032	0.094	0.059	0.618
d 25^2^		0.304	0.342	0.351	0.016	0.421	0.219	0.66
d 39		0.18	0.193	0.18	0.0089	0.79	0.793	0.716
NO, μmol/L	Serum							
d 23		5.26	6.02	6.06	0.394	0.574	0.421	0.528
d 25		3.3	4.31	4.48	0.33	0.308	0.149	0.561
d 39		3.24	3.84	3.14	0.358	0.713	0.915	0.42
TBARS, mmol MDA/mL	Plasma							
d 23		14.7	14.4	14.2	0.26	0.785	0.492	0.951
d 25		14.8	14.3	13.2	0.28	0.057	0.02	0.592
d 39		17.1	15.7	14.9	0.42	0.076	0.025	0.698
GPx, U/mL	Plasma							
d 23		0.85	0.82	0.92	0.051	0.687	0.547	0.537
d 25		0.81	0.84	0.86	0.032	0.815	0.527	0.942
d 39		1	0.97	1.09	0.033	0.291	0.239	0.311
SOD, % inhibition	Plasma							
d 23		79.9	84.1	80.4	2.24	0.709	0.926	0.413
d 25		84.8	81.3	84.1	1.86	0.721	0.883	0.43
d 39		88	79.5	87.1	2.18	0.236	0.847	0.093
GSH, μmol/mL	Erythrocytes							
d 23		0.91	1.07	1.25	0.066	0.103	0.035	0.93
d 25^2^		0.59	0.59	0.7	0.026	0.088	0.052	0.303
d 39		0.50^b^	0.64^ab^	0.67^a^	0.031	0.037	0.018	0.311
GSSG, μmol/mL	Erythrocytes							
d 23		0.016	0.017	0.023	0.002	0.322	0.167	0.56
d 25		0.009	0.01	0.009	0.0005	0.921	0.886	0.707
d 39		0.009	0.011	0.01	0.0006	0.22	0.564	0.102
GSSG/GSH	Erythrocytes							
d 23		0.018	0.015	0.018	0.0011	0.6	0.887	0.321
d 25		0.016^ab^	0.017^a^	0.013^b^	0.0006	0.027	0.054	0.049
d 39		0.017	0.017	0.015	0.0007	0.454	0.446	0.446

Broilers were fed a wheat-soybean starter diet from d 0 to 10, a grower diet from d 10 to 21 and a finisher diet from d 21 to 39. The chronic cyclic heat stress model was implemented from d 24 till 39.

a,b Values with different superscripts within a row are significantly different at *P* < 0.05 (*n* =12). Abbreviations: GPx, glutathione peroxidase; GSH, glutathione; GSSG, glutathione disulfide; HSP70, heat shock protein 70; MDA, malondialdehyde; MSM, methyl sulfonyl methane; NO, nitric oxide; SOD, superoxide dismutase; TBARS, thiobarbituric acid reactive substances; T3, triiodothyronine; T4, thyroxine.

1 Many values were below detection limit of 0.42 μg/dL, hence statistical analysis was not done.

2 Variable was significantly negatively influenced by time after starting heat stress.

With regard to the antioxidant status in blood (Table 6), NO was not different across treatments at any sampling day. Further, interesting changes in TBARS were found. On both d 25 and 39, a linear decrease was found when feeding MSM (both *P* < 0.05). Remarkable is also the large increase in TBARS from d 25 to 39 in Ctrl birds (+2.3 nmol/mL). The activity of the antioxidant enzymes GPx and SOD were not changed by diet. In contrast, GSH was on all days increased by adding MSM to the diet, that is, a linear increase on d 23 and 39 (both *P* < 0.05), and a trend for linear increase on d 25 (*P* = 0.052). The birds fed 2 g/kg MSM diet possessed significant higher GSH content in erythrocytes as compared to Ctrl broilers. GSSG in erythrocytes was not affected by the supplementation of MSM. Consequently, the ratio GSSG-to-GSH was notably decreased by 2 g/kg MSM addition when compared to Ctrl (*P* < 0.05).

For some cases, the time after starting HS that day affected linearly the response for the outcome (Supplementary Figure 2). In particular, T3, T3/T4, and GSH were all decreasing when time advanced irrespective of treatment on sampling d 25 (all *P* < 0.05).

### Antioxidant Status in Liver and Breast Muscle

As illustrated in Table 7, only GPx activity was different across treatments at d 23 in liver. A linear decrease was demonstrated (*P* < 0.05) as the supplementation level increases, though differences are small. Furthermore, the decline in GSH levels and the increases in the ratio GSSG-to-GSH were noticed in aging birds. In breast muscle, on d 23, a linear decrease in TBARS upon supplemental MSM was found (*P* < 0.05) and not on the other days (Table 8). When compared to Ctrl, the supplementation with 1 g/kg and 2 g/kg MSM significantly decreased the content of TBARS (*P* < 0.05). GSH levels increased with age, from 1.03–1.10 to 1.39–1.56 μmol/g (d 23–39). Interestingly, GSH was elevated linearly by MSM on d 25 (*P* < 0.05) and a trend suggests the same for d 39 (*P* = 0.066).

**Table 7:** Dietary effect on parameters in liver in male broilers subjected to chronic cyclic heat stress.

Item	Ctrl	1 g/kg MSM	2 g/kg MSM	SEM	*P-*value		
					Model	Linear	Quadratic
TBARS, mmol MDA/g							
d 23	46	43.9	44.6	0.7	0.477	0.447	0.343
d 25	51	48.6	49.1	0.7	0.323	0.245	0.323
d 39	49.3	46.4	48	0.97	0.355	0.916	0.44
GPx, U/g							
d 23	17.9	17	16.9	0.2	0.091	0.043	0.395
d 25	18.2	17.8	17.9	0.22	0.691	0.569	0.514
d 39	18.2	17.5	18.6	0.29	0.303	0.535	0.163
SOD, U/g							
d 23	307	332	317	6.7	0.303	0.545	0.156
d 25	274	292	289	6.7	0.507	0.342	0.489
d 39	358	356	346	6.5	0.637	0.686	0.636
GSH, μmol/g							
d 23	5.07	4.69	5.04	0.107	0.279	0.909	0.114
d 25^a^	4.06	4.26	4.14	0.105	0.676	0.697	0.578
d 39	3.82	3.74	3.94	0.091	0.676	0.574	0.497
GSSG, μmol/g							
d 23	0.081	0.077	0.076	0.0014	0.36	0.198	0.52
d 25	0.076	0.07	0.069	0.002	0.303	0.144	0.575
d 39	0.099	0.093	0.097	0.0025	0.688	0.789	0.411
GSSG/GSH							
d 23	0.016	0.016	0.015	0.0004	0.473	0.899	0.227
d 25	0.018	0.017	0.017	0.0006	0.559	0.494	0.398
d 39	0.026	0.027	0.025	0.0011	0.8	0.746	0.563

Broilers were fed a wheat-soybean starter diet from d 0 to 10, a grower diet from d 10 to 21 and a finisher diet from d 21 to 39. The chronic cyclic heat stress model was implemented from d 24 till 39.

Abbreviations: GPx, glutathione peroxidase; GSH, glutathione; GSSG, glutathione disulfide; MDA, malondialdehyde; MSM, methyl sulfonyl methane; SOD, superoxide dismutase; TBARS, thiobarbituric acid reactive substances.

a The time after starting heat stress that day affected linearly the response for the outcome.

**Table 8:** Dietary effect on parameters in breast muscle in male broilers subjected to chronic cyclic heat stress.

Item	Ctrl	1 g/kg MSM	2 g/kg MSM	SEM	*P-*value		
					Model	Linear	Quadratic
TBARS, mmol MDA/g							
d 23	5.72^a^	4.72^b^	4.50^b^	0.185	0.011	0.005	0.28
d 25	4.01	4.6	4.5	0.138	0.18	0.151	0.222
d 39	5.42	5.23	5.57	0.246	0.881	0.808	0.662
GPx, U/g							
d 23	0.4	0.39	0.38	0.008	0.763	0.483	0.839
d 25	0.42	0.42	0.44	0.01	0.471	0.325	0.481
d 39	0.45	0.46	0.5	0.022	0.274	0.133	0.605
SOD, U/g							
d 23	30.3	29.7	32.6	1.32	0.641	0.485	0.53
d 25	28.6	27.3	28.1	1.14	0.893	0.862	0.658
d 39	29.1	27.4	26.2	0.87	0.416	0.19	0.886
GSH, μmol/g							
d 23	1.03	1	1.1	0.024	0.183	0.189	0.181
d 25^1^	1.12^b^	1.30^ab^	1.39^a^	0.04	0.02	0.006	0.61
d 39	1.39	1.46	1.56	0.039	0.175	0.066	0.835

Broilers were fed a wheat-soybean starter diet from d 0 to 10, a grower diet from d 10 to 21 and a finisher diet from d 21 to 39. The chronic cyclic heat stress model was implemented from d 24 till 39.

1 The time after starting heat stress that day affected linearly the response for the outcome.

a,b Values with different superscripts within a row are significantly different at *P* < 0.05 (*n* = 12). Glutathione disulfide was below detection limit (0.05 μmol/g). Abbreviations: GPx, glutathione peroxidase; GSH, glutathione; MDA, malondialdehyde; MSM, methyl sulfonyl methane; SOD, superoxide dismutase; TBARS, thiobarbituric acid reactive substances.

## DISCUSSION

In this study, we showed that supplementing MSM improved performances of broilers in the grower period, likely with carry-over effects to the finisher phase fostering tolerance to heat and consequently lowering mortality when HS was applied. Tolerance to HS was evidenced by reduced rectal temperature and panting frequency and improved oxidative status, most notable in blood. MSM altered color slightly and enhanced oxidative stability of breast meat.

### Methyl Sulfonyl Methane Promotes Feed Efficiency in Grower Phase and Supports Tolerance to Heat in Finisher Phase

Most notable finding was the largely improved performance in the grower phase. BW at d 21 and ADG showed linear increases with higher inclusion of MSM in the diet, and consequently remarkably improved feed efficiency. These results agree with other studies on meat-type domestic birds grown under thermo-neutral conditions (Supplementary Table 1). Liu and Zhou (2008) found that a diet with 0.250 g/kg MSM could increase ADG and feed efficiency in meat ducks. A higher dosage (0.5–2.0 g/kg MSM) was applied by Jiao et al. (2017) resulting in linearly increased body weight gain and reduced F:G during the period d 1 to 29 in broilers. For example, in that study F:G was 1.48 and 1.45 for MSM at 0 and 2 g/kg, respectively. Remarkably, this is a similar improvement in F:G as we observed for starter + grower period (d 0–21) when broilers were given either 1 or 2 g/kg MSM. Dietary supplementation with 3 g/kg MSM also increased weight gain and decreased F:G of ducks in finisher phase (d 22–42) (Yan et al., 2020). On the contrary, dietary MSM (0.3 g/kg MSM) for Cherry Valley male ducks did not affect ADG, ADFI, and feed efficiency (Hwang et al., 2017), and also Rasheed et al. (2020a) could not demonstrate performance improvements in broilers fed 0.5 g/kg MSM during an oxidative challenge. It appears, at least with regard to broilers, that dosages in the range of our study are required to elicit a significant beneficial performance response. Nonetheless, the finding that in our study only in the grower phase MSM exhibited this response might be due to diet formulation. Indeed, diets were formulated according to the ideal amino acid profile with digestible lysine as reference. Digestible lysine dropped substantially between starter, that is, 1.24%, and grower, that is, 1.05%, diet. The subsequent reduction in finisher diets was smaller (0.99 vs. 1.05%). It is plausible that effects of MSM could be more accentuated in low crude protein diets, more specifically when formulated for lower amounts of essential amino acids. The interaction between crude protein content and MSM needs further investigation.

Heat stress leads to negative effects on performance due to the reduction in feed consumption and nutrient absorption (Wiernusz and Teeter, 1996; Habashy et al., 2017) and oxidative stress (Akbarian et al., 2016). As such, it was expected that MSM would restore the decline of performance induced by HS. However, the current results do not support this notion as neither final BW nor F:G in finisher phase was improved by MSM as compared to Ctrl. HS during the 6 h of high temperatures daily was evidenced by high rectal temperatures and excessive panting. The BW at d 39 across treatments was 3,083 g. Yet, this may indicate high performance despite 15 d of chronic cyclic HS. No thermo-neutral control was included in the study, however, based on gains in starter + grower and the outcomes of the meta-analysis by Flécher et al. (2019) we were able to estimate a final BW as if the HS model would not have been implemented of 3,272 g (calculations upon request). Thus, the implementation of the chronic cyclic HS model reduced final BW by 189 g or 5.8%, equivalent to a reduction of 9.1% for ADG in finisher period. Moreover, this meta-analysis suggested that in chronic cyclic HS models the reduction in growth mostly comes from deterioration of feed efficiency, whereas in chronic constant cyclic HS models (constant high temperature throughout the 24 h/d) this is mostly explained by reduced FI. In this respect, the F:G ratio in finisher period should be worsened more than the estimated 9.1% for ADG in finisher period. Indeed, F:G here was between 1.69 and 1.72. Again, according to the meta-analysis, we estimated the F:G ratio to be between 1.46 and 1.50 in thermo-neutral conditions resulting in an estimated average reduction of 15.3% (calculations upon request). One can argue that a reduction in growth inflicted by HS with 9.1% is not huge, and probably opposite to practical observations. Indeed, in the current HS model, birds compensate heavily in the low-temperature period of the day, particularly regarding FI. However, the metabolic outcome from the 6 h high temperature per day might determine the capacity to compensate during the rest of the day.

A mortality of 6.3% for Ctrl is commonly seen when applying this chronic cyclic HS model in our facility (e.g., Majdeddin et al., 2020). Reductions in mortality as seen for 1 (−1.6%) and 2 (−3.2%) g/kg MSM are rarely found, thus emphasizing the importance of the numerical reductions here as further illustrated in the survival curves. Indeed, to find significance for these types of data more birds are needed. Interestingly, mortality in Ctrl occurred mostly in the beginning of the chronic cyclic HS model, with peak mortality on d 28, whereas in the 2 g/kg MSM group dead birds were found mostly in the second half of this period. It may suggest that carry-over effects from grower phase resulted in higher tolerance in the first days of HS. This is further substantiated by the reduction in panting frequency on d 24 and rectal temperature on d 25 by 2 g/kg MSM. In line with this, FI_HS_ for d 25 and 26 showed a trend for linear effect by incremental levels of MSM, which might indicate that supplemented birds suffered less from heat increment induced by feed consumption. It is known that stressful conditions reduce circulating thyroid hormones to preserve body temperature via altered energy metabolism (Sohail et al., 2010). Further, thyroid hormones play an important role in thermoregulation of avian species. A suppression in the thyroid axis is believed to be associated with HS, and the conversion of T4 to T3, the latter biologically more active, is decreased, leading to a lower level of T3 in broilers (Oke et al., 2017). Accordingly, significantly reduced blood T3 concentrations have been reported in broilers exposed to chronic cyclic HS (Zaglool et al., 2019). In the current study, under the thermal-neutral condition, that is, d 23, the day prior to the initiation of HS, dietary MSM treatments contributed to the decrease in serum T3, implying a decrease in the metabolic rate. This reduced T3 may have prevented undesirable catabolic effects in the first days of HS and explain partly the lower panting frequency and rectal temperature. However, as indicated above T3 dropped dramatically during the HS period, particularly in Ctrl. On d 39, a trend suggests that T3 was linearly elevated by MSM. It may underline a better metabolic adaption to chronic HS, supported by the observations that at the end of the study the diets with graded levels of MSM resulted in a linear reduction of rectal temperatures and a trend for linear increase of FI_HS_. Meanwhile, exposure to high ambient temperatures also increases the synthesis of HSP70, which is vital to stress recovery and is responsible for repairing damaged cells (Xie et al., 2014) and inhibiting oxidation and apoptosis (Xing et al., 2015). This is obviously true when comparing HSP70 plasma concentrations before HS and after 15 d of HS (doubled to quadrupled values), yet the acute HS (d 25) showed a drop. To conclude here, it is fair to postulate that MSM supported tolerance to early heat and stimulated an adaptive response to chronic HS.

### Methyl Sulfonyl Methane Has Minor Effects on Carcass and Breast Meat Characteristics but Supports Oxidative Stability of Breast Meat

As far as carcass yields and breast meat characteristics are concerned, there were no differences in the relative weight of abdominal fat, carcass, breast, thigh, and drumstick in the current study. In line with our observations, Yan et al. (2020) reported that diets with 1.5 or 3.0 g/kg of MSM did not influence the relative weight of carcass, breast, and abdominal fat in Pekin ducks and no apparent effects of MSM (0.5–2 g/kg) on the relative weight of breast and abdominal fat were noticed in broilers (Jiao et al., 2017). Regarding the quality of breast meat, it is well-known that WHC plays an important role in raw and processed meat products through affecting the sensory characteristics and nutritional value of meat. In contrast to our findings, MSM-fed poultry exhibited higher WHC and lower drip loss (Hwang et al., 2017; Jiao et al., 2017; Yan et al., 2020). Drip and press loss were not affected in the current study which can be explained by the lack of effect on pH 24 h. It is well established that pH fall postmortem has an association with WHC of meat (Gou et al., 2002). Jiao et al. (2017) did not observe a positive effect of MSM (0.5–2 g/kg) on pH or WHC in broilers, yet only drip loss was positively affected. MSM feeding caused higher redness (a*) of breast meat in broilers and ducks (Jiao et al., 2017; Yan et al., 2020) and of loin in pigs (Lee et al., 2009). These findings were explained by increased Fe-haem binding and delayed metmyoglobin formation postmortem. This was not observed in the present study, although a* was consistently numerically higher in MSM groups. However, the diet supplemented with 1 g/kg MSM decreased yellowness. Interestingly, in this study, a trend indicates that the TBARS values were linearly reduced after the supplementation of MSM, which was consistent with previous findings in ducks (Hwang et al., 2017; Yan et al., 2020) and finishing pigs (Lee et al., 2009). The reduced TBARS level may imply that MSM exerts potent antioxidant activities to scavenge free radicals. Further, we found that the birds that received 2 g/kg MSM diets had higher incidence of moderate white striations, implicating that the fillets might have an increase in fat content and a corresponding decrease in protein percentages (Petracci et al., 2014). This could result in changes in nutritive value of end meat products and attract the attention of customers. It was reported that breast meat with white striping had lower WHC and tenderness since degeneration of muscle fibers results in decreased myofibrillar and sarcoplasmic proteins (Petracci et al., 2013; Mudalal et al., 2014). WHC and drip and press loss did not change in our study which might indicate that the impact of 2g/kg MSM on incidence of white striations has minor consequences and might not be perceivable by the consumer.

### Effects of Methyl Sulfonyl Methane Are Mediated by Enhanced Antioxidant Defenses

The effects of MSM on antioxidant system were quantified since HS could induce oxidative stress in broilers (Akbarian et al., 2016). MDA is derived from the cleavage of polyunsaturated fatty acids by ROS and is used as standard in the TBARS method. It is the main final product of the lipid peroxidation processes in cells (Del et al., 2005). In the present study, MSM administration linearly decreased TBARS in plasma at d 25 and 39, corresponding to acute and chronic HS, respectively. This observation is in line with results of previous studies showing that supplementation of MSM (3 g/kg) decreased TBARS levels in serum of Cherry Valley male ducks (Yan et al., 2020) and the works of Rasheed et al. (2020a,b) employing oxidized oil and *Eimeria* challenges in broilers, fed 0.5 and 4 g/kg MSM, respectively. On the mechanism, MSM has been shown to repress the transcriptional activity of NF-κB, which was confirmed in chickens by Miao et al. (2022), and the expression and activities of STAT, which altogether may lower the generation of ROS, plausibly explaining the lower levels of TBARS. Furthermore, several organosulfur compounds have shown to be electrophilic toward redox sensitive cysteine residues in proteins, and thereby take action in the redox regulation pathway. Importantly, Nrf2 activation is controlled by redox regulation, as Nrf2 release form the Kelch-like ECH-associated protein 1 (**Keap1**) complex is promoted by critical thiols within Keap1. Nrf2 is then liberated and diffuses to the nucleus where it binds to the antioxidant response element (**ARE**). The ARE is found in the promoter region of genes encoding for several antioxidant enzymes (e.g., SOD). Also the gene regulation of several GSH-related enzymes, like GPx, glutathione reductase (**GR**) and GCL, is under control of this redox sensitive system (Egbujor et al., 2022). MSM has been described as a organosulfur compound with the ability to activate Nrf2 release by redox regulation, and hence induce the transcription of antioxidant genes (Liang et al., 2011; Kim et al., 2015). For example, a potential increase GCL, catalyzing the rate-limiting step in GSH synthesis, could have explained the increased GSH levels in erythrocytes and breast muscle with increasing MSM dose. However it is questionable if this occurred in the current experiment, as MSM did not result in an increased SOD and GPx activities in any of the measured tissues, and linearly reduced hepatic GPx activity before HS was found. Next, it was striking that GSH levels in erythrocytes were linearly increased at all time points (d 23, 25, and 39). Moreover, at d 25, GSH content in erythrocytes was negatively influenced by time after starting HS. In addition, we observed that GSH levels in erythrocytes were remarkably reduced during HS (d 25 and 39 vs. d 23 in Table 6) congruent to previous studies in our group (unpublished data). Both observations might be caused by higher needs in other tissues such as small intestine, lungs, and breast, or by depressed synthesis of GSH and regeneration of GSH via salvation pathway as HS advances. Regarding the latter, the ratio GSSG-to-GSH in erythrocytes from d 25 to 39 tremendously increased. This suggest that salvation, that is, the reduction of GSSG to GSH catalyzed by GR is hampered leading to redox disturbances. Also striking is the increase of GSH levels in breast muscle with age; it means that large amounts of GSH are needed to supply muscle. The fact that MSM is able to stimulate GSH synthesis is therefore even more appealing. Erythrocytes can contribute significantly to the extracellular pool of GSH cooperating with liver, another main tissue for GSH synthesis, to replenish tissues during inter-organ GSH metabolism (Giustarini et al., 2008). That can also be seen in breast muscle in our study, MSM caused higher GSH concentrations at d 25 and 39. Substantial evidence points at the pivotal role of redox molecules such as GSH, which as thiol-containing compound is the main intracellular antioxidant and plays an important role in the protection against free radicals and ROS (Dröge, 2002). The GSH redox system offers the opportunity to efficiently buffer undesirable oxidation reactions and is converted to GSSG when it is oxidized. Cells tightly regulate these oxidation and reduction reactions and strive to keep GSH in a predominantly reduced status. By the law of Nernst, this status is expressed by its half-cell redox potential (GSH/GSSG Eh) (Schafer and Buettner, 2001). In this regard, MSM is a source of sulfur and may provide organic sulfur to the synthesis of GSH (Butawan et al., 2017), but this role of MSM can be debated if no significant gut microbial metabolization of MSM takes place. In ovo sulfur amino acid injection (methionine plus cysteine) in embryonated eggs exposed to HS increased GPx gene expression and GSH/GSSG ratio of serum, liver, small intestine, and pectoral muscle, whereas decreased HSP70 transcription and corticosterone content were seen in newly hatched broilers (Elnesr et al., 2019). Analogously, the results of a kidney injury model in rats confirmed that animals treated with 4 g/kg MSM showed significantly increased GSH concentration in rats (Al Laham, 2018). Administration of MSM reduced the depletion of GSH caused by oxidative stress following exercise in human (Nakhostin-Roohi et al., 2013) and horses (Marañón et al., 2008). Next to the upregulation of GSH synthesis it may be hypothesized that MSM acts as an alternative source of sulfate and hitherto spares cysteine for synthesis of GSH, but this remains to be confirmed. Taken together, these findings indicate that dietary MSM supplementation could improve the antioxidant status of broilers by enhancing GSH levels and protect heat-stressed birds from oxidative stress.

## CONCLUSIONS

HS is an environmental stressor challenging poultry production and known to inflict oxidative stress. In this study, it was confirmed that a dose of 1 and 2 g/kg MSM exerts a positive effect on growth and feed efficiency in grower phase of broilers, with likely carry-over effects on heat-stressed broilers in the finisher phase. MSM enhanced heat tolerance and indications suggest reduced mortality during HS. Oxidative stability of breast meat was improved due to antioxidant properties of MSM. In this respect, MSM-fed birds showed higher GSH levels in erythrocytes and breast muscle, likely due to stimulation of its synthesis.

## Appendix Supplementary materials

Image, application 1

## Footnotes

Supplementary material associated with this article can be found in the online version at doi:10.1016/j.psj.2022.102321.
