# Supplementary material for: Effect of supplemental methyl sulfonyl methane on performance, carcass and meat quality and oxidative status in chronic cyclic heat-stressed finishing broilers
Source: Poult Sci. 2022 Nov 9;102(2):102321. doi: 10.1016/j.psj.2022.102321 (PMC9763687; doi:10.1016/j.psj.2022.102321)
Supplement: Supplementary file 1 [file mmc1.docx]

**Supplementary Table 1.** Overview of studies on the effects of methyl sulphonyl methane (**MSM**) on meat producing poultry.

| Species | Treatments | Results | Reference |
| --- | --- | --- | --- |
| Meat ducks (Tianfu) | 14-day-old meat ducks were fed basal diet, or basal diet with either 0.150 g/kg plant extracts, 0.150 g/kg cysteamine, or 0.250 g/kg MSM for 35 d. | 1) MSM could increase average body weight gain and feed efficiency during 14-28 d and 14-35 d.  2) MSM could improve slaughter characteristics of the ducks such as increased muscle ratio and decreased abdomen fat ratio in the carcass. | Liu and Zhou (2008) |
| Meat ducks  (Cherry Valley) | Male ducklings were fed diets supplemented with 0.3 g/kg MSM alone and with a combination of 0.3 g/kg MSM and 5 g/kg oriental herbal medicine residue (**OHMR**) for 42 d. | 1) Supplementing the diet with OHMR and MSM resulted in a significant decrease in mortality rate and serum total cholesterol (**TC**) concentration, and in an increase in the antioxidant enzyme activities of superoxide dismutase (**SOD**) and catalase in duck breast muscle (*Musculus pectoralis*) by 3 and 6 weeks.  2) MSM alone or combined with OHMR increased the concentrations of crude protein and sulfur content, and water-holding capacity, while it decreased percentage moisture loss and thiobarbituric acid reactive substances (**TBARS**) during cold storage. | Hwang et al. (2017) |
| Broilers  (Ross 308) | 1-d-old male broilers were assigned to basal diet with 0, 0.5, 1.0, or 2.0 g/kg MSM for 29 d | 1) MSM linearly increased body weight gain and decreased feed to gain ratio (**F:G**) during 1-29 d.  2) Redness (a*) was increased linearly in broilers fed MSM diets, and drip loss was decreased linearly in broilers fed MSM diets on d 3, 5, and 7.  3) MSM diets linearly increased and reduced the abundance of *Lactobacillus* and *E. coli* in excreta. | Jiao et al. (2017) |
| Broilers  (Ross 308) | 15-day-old male broilers were administered a single oral dose of MSM at 0, 50, 100, 300, 1,000, or 2,000 mg/kg BW (Study 1).  3-d-old chicks were allotted to either daily oral gavage of either 0 or 1, 500 mg/kg BW of MSM for d 21 consecutively (Study 2). | 1) In study 1, plasma MSM concentrations were below 167 μg/mL in birds receiving up to 300 mg/kg BW and were significantly higher in birds receiving 1,000 or 2,000 mg/kg BW. The latter two treatments increased lymphocyte and decreased heterophil counts at 8 h and decreased hematocrit at 48 h.  2) In study 2, growth performance variables were unaffected by MSM. Birds fed MSM diet had decreased liver enzyme concentrations at d 7 and 21 and decreased glucose and phosphorus at d 7. | Rasheed et al. (2019) |
| Broilers  (Ross 308) | Day-old male broilers were allocated to 4 dietary treatments: fresh soybean oil-no MSM, fresh soybean oil-MSM, oxidized soybean oil-no MSM, oxidized soybean oil-MSM for 25 d. MSM was fed at 0.5 g/kg. | 1) MSM did not affect growth equally across time points.  2) In the presence of oxidized oil, MSM reduced plasma TBARS at d 21. Irrespective of dietary oil type, groups supplemented with MSM showed higher plasma total antioxidant capacity (**TAC**) at d 7, liver glutathione peroxidase (**GPx**) activity at d 21, and liver reductase activities activity at d 7 compared with groups not receiving MSM. | Rasheed et al. (2020a) |
| Broilers  (Ross 308) | Day-old male chicks were allocated to 5 treatments: sham-inoculated (uninfected) chickens fed control diet (**UCON**), *Eimeria*-infected chickens fed control diet (**ICON**), and *Eimeria*-infected chickens fed control diet supplemented with 287 U/ton of from dried egg product (**I-DEP**) for suppling IL-10-neutralizing antibody, 4 g/kg MSM, or their combination (**I-DEP-MSM**). | 1) Overall (d 0-28) growth performance parameters were not influenced by either infection or dietary supplementation with MSM or DEP. However, birds in I-DEP-MSM showed improved ADG during study d 7 to 14.  2) MSM supplementation reduced TBARS (d 21 and 28), both MSM and DEP improved the TAC (d 21) in the plasma of infected birds.  3) Expression of cecal inflammatory cytokines (interleukin-10 (**IL**-10), IL-1β, and interferon-γ) was not affected by MSM, DEP, or their combination. | Rasheed et al. (2020b) |
| Meat ducks  (Peking) | 1-d-old ducklings were randomly allotted to a corn-soybean meal-based diet supplemented with 0, 1.5, and 3.0 g/kg MSM for 42 d. | 1) Diet with 3 g/kg MSM increased body weight gain and lowered feed-to-gain ratio during 22-42 d, as well improved body weight gain and final BW during 1-42 d as compared to control and 1.5 g/kg MSM groups. 2) Higher SOD and GPx, TAC, and concentrations of IL-2 and IL-6, whereas lower serum malondialdehyde (**MDA**), interferon gamma (**IFN-γ**), and tumor necrosis factor (**TNF**)-α levels were observed in 3 g/kg MSM as compared to control treatment.  3) The supplementation of MSM increased water-holding capacity and redness (a*) and decreased values for TBARS and drip loss, moreover ducks in the 3 g/kg MSM treatment group had higher pH_24h_ than those in the control group. | Yan et al. (2020) |
| Chickens  (White Leghorn) | Day-old birds were randomly divided into control group, *Mycoplasma gallisepticum* (**MG**) group, MG group treated with MSM (0.25, 0.5 and 1 g per kg BW) and MSM alone treated group (0.5 g per kg BW) for 7 d. MG infected group chickens were inoculated with MG strain Rlow (1 × 10^9^ CCU/mL) in the left air sacs at d 7. | 1) MSM treatment significantly ameliorated oxidative stress, partially alleviated the abnormal morphological changes, and reduced MG colonization.  2) MSM reduced the mRNA expression of pro-inflammatory cytokines-related genes and decreased the number of death cells under MG infection. 3) MSM suppressed the nuclear factor-kappa B (**NF-κB**) and extracellular signal-related kinases (**ERK**)/Jun amino terminal kinases (**JNK**)-mitogen-activated protein kinases (**MAPK**) pathway in trachea. | Miao et al. (2022) |

**References**

Hwang, J.W., S.H. Cheong, Y.S. Kim, J.W. Lee, B.-I. You, S.H. Moon, B.T. Jeon, and P.-J. 2017. Effects of dietary supplementation of oriental herbal medicine residue and methyl sulfonyl methane on the growth performance and meat quality of ducks. Anim. Prod. Sci. 57: 948-957.

Jiao, Y., J.H. Park, Y.M. Kim, and I.H. Kim. 2017. Effects of dietary methyl sulfonyl methane (MSM) supplementation on growth performance, nutrient digestibility, meat quality, excreta microbiota, excreta gas emission, and blood profiles in broilers. Poult. Sci. 96: 2168-2175.

Liu, H.F., and A.G. Zhou. 2008. Effects of plant extracts, cysteamine and methylsulfonylmethane on productive performance and slaughter characteristics in meat ducks. Nat. Prod. Res. Dev*.* 20: 302-306.

Miao, Y., D. Niu, Z. Wang, J. Wang, Z. Wu, J. Bao, X. Jin, R. Li, M. Ishfaq, and J. Li. 2022. Methylsulfonylmethane ameliorates inflammation via NF-kappa B and ERK/JNK-MAPK signaling pathway in chicken trachea and HD11 cells during *Mycoplasma gallisepticum* infection. Poult. Sci. 101: 101706.

Rasheed, M.S.A., M.L. Oelschlager, B.N. Smith, L.L. Bauer, R.A. Whelan, and R.N. Dilger. 2019. Toxicity and tissue distribution of methylsulfonylmethane following oral gavage in broilers. Poult. Sci. 98:4972-4981.

Rasheed, M.S.A., M.L. Oelschlager, B.N. Smith, L.L. Bauer, R.A. Whelan, and R.N. Dilger. 2020a. Dietary methylsulfonylmethane supplementation and oxidative stress in broiler chickens. Poult. Sci. 99: 914-925.

Rasheed, M.S.A., U.P. Tiwari, J.C. Jespersen, L.L. Bauer, and R.N. Dilger. 2020b. Effects of methylsulfonylmethane and neutralizing anti-IL-10 antibody supplementation during a mild *Eimeria* challenge infection in broiler chickens. Poult. Sci. 99: 6559-6568.

Yan, H.L., S.C. Cao, Y.D. Hu, H.F. Zhang, and J.B. Liu. 2020. Effects of methylsulfonylmethane on growth performance, immunity, antioxidant capacity, and meat quality in Pekin ducks. Poult. Sci. 99:1069-1074.

**Supplementary Table 2.** Analysed nutrient composition of corn-soybean based basal diets^1^.

| Item | Starter (d 0-10) | | | Grower (d 10-21) | | | Finisher (d 21-39) | | |
| --- | --- | --- | --- | --- | --- | --- | --- | --- | --- |
|  | Ctrl | 1 g/kg MSM | 2 g/kg MSM | Ctrl | 1 g/kg MSM | 2 g/kg MSM | Ctrl | 1 g/kg MSM | 2 g/kg MSM |
| Dry matter^†^ | 89.5 | 89.8 | 89.9 | 89.4 | 89.8 | 90.1 | 89.4 | 89.4 | 89.7 |
| Metabolic energy^†^, kcal/kg | 2885 | 2915 | 2898 | 2973 | 2984 | 2958 | 3039 | 3051 | 3054 |
| Ether extract^†^ | 5.6 | 5.6 | 5.5 | 6.3 | 6.2 | 6.0 | 7.2 | 7.1 | 7.1 |
| Ash^†^ | 5.8 | 5.6 | 5.6 | 5.1 | 5.0 | 5.0 | 4.6 | 4.8 | 4.7 |
| Starch^†^ | 36.9 | 37.5 | 37.2 | 40.4 | 40.5 | 40.4 | 41.8 | 42.3 | 41.7 |
| Phosphorus^†^ | 6.5 | 6.1 | 6.1 | 5.5 | 5.4 | 5.1 | 4.7 | 4.9 | 4.7 |
| Crude protein | 22.8 | 22.7 | 22.7 | 20.0 | 19.8 | 19.8 | 18.7 | 18.3 | 18.8 |
| Lysine | 1.40 | 1.39 | 1.39 | 1.18 | 1.18 | 1.17 | 1.10 | 1.10 | 1.10 |
| Methionine + cysteine | 1.02 | 1.03 | 1.00 | 0.86 | 0.86 | 0.86 | 0.81 | 0.81 | 0.81 |
| Threonine | 0.94 | 0.94 | 0.95 | 0.81 | 0.80 | 0.80 | 0.74 | 0.74 | 0.75 |
| Valine | 1.09 | 1.09 | 1.09 | 0.92 | 0.92 | 0.92 | 0.86 | 0.87 | 0.87 |
| Arginine | 1.44 | 1.43 | 1.45 | 1.22 | 1.23 | 1.23 | 1.13 | 1.14 | 1.13 |
| Isoleucine | 0.92 | 0.92 | 0.91 | 0.78 | 0.79 | 0.78 | 0.73 | 0.73 | 0.73 |
| Leucine | 1.60 | 1.58 | 1.59 | 1.38 | 1.38 | 1.38 | 1.29 | 1.29 | 1.29 |
| Lysine supplemental | 0.28 | 0.28 | 0.28 | 0.26 | 0.25 | 0.25 | 0.26 | 0.26 | 0.25 |
| Methionine supplemental | 0.33 | 0.34 | 0.34 | 0.24 | 0.24 | 0.24 | 0.22 | 0.22 | 0.22 |
| Threonie supplemental | 0.16 | 0.17 | 0.17 | 0.14 | 0.14 | 0.14 | 0.13 | 0.13 | 0.13 |
| Valine supplemental | 0.09 | 0.09 | 0.09 | 0.05 | 0.05 | 0.05 | 0.05 | 0.05 | 0.05 |

^1^ Nutrients were analysed by near infrared spectroscopy, ^†^, or wet chemistry, and given in %, unless otherwise stated (Evonik Operations; Hanau-Wolfgang, Germany).

**Supplementary Table 3.** Effect of dietary supplementation with 1 and 2 g/kg of methyl sulphonyl methane (**MSM**) on body weight (**BW**) and rectal temperature in sampled male broilers subjected to chronic cyclic heat stress.

| Item | Ctrl | 1 g/kg MSM | 2 g/kg MSM | SEM | *P-*value | | |
| --- | --- | --- | --- | --- | --- | --- | --- |
|  |  |  |  |  | Model | Linear | Quadratic |
| BW, g  d 23  d 25  d 39  Rectal temperature, °C  d 23  d 25  d 39 | 1341  1535  3071  41.3  42.8^a^  42.7 | 1361  1544  3097  41.3  42.5^b^  42.6 | 1352  1536  3068  41.1  42.3^b^  42.3 | 5.9  6.3  15.0  0.06  0.07  0.09 | 0.421  0.827  0.638  0.358  0.005  0.263 | 0.474  0.957  0.846  0.183  0.006  0.127 | 0.271  0.542  0.500  0.604  0.544  0.571 |

Broilers were fed a wheat-soybean starter diet from d 0 to 10, a grower diet from d 10 to 21 and a finisher diet from d 21 to 39. The chronic cyclic heat stress model was implemented from d 24 till 39. On d25 and d39, broilers were sampled between 3 and 6-7 h of high temperatures on the respective day. Values with different superscripts within a row are significantly different at *P* < 0.05 (n =12).

SEM = standard error of the mean.


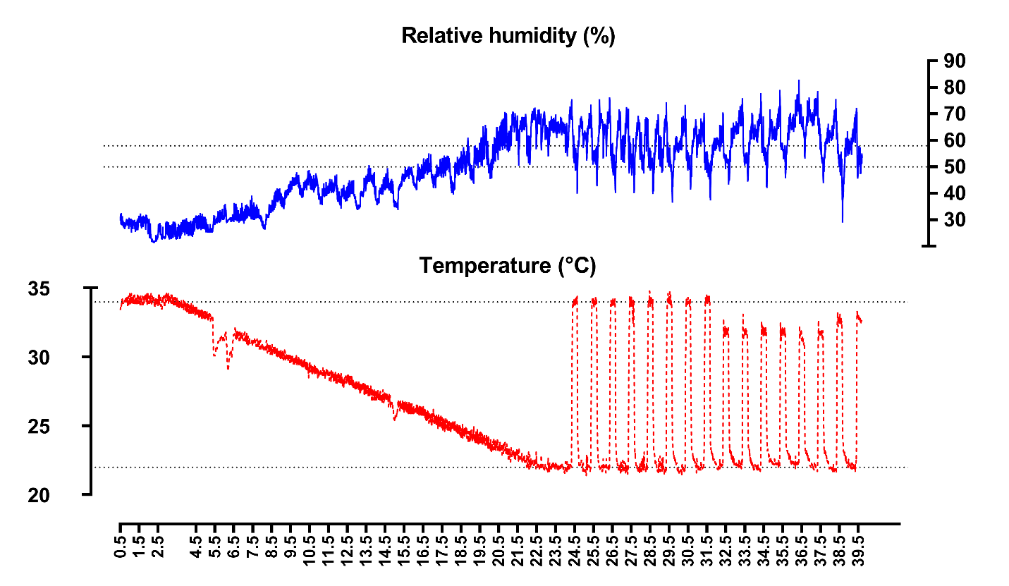


**Supplementary Figure 1.** Relative humidity (blue line, right Y-axis) and temperature (red line, left Y-axis) from d 0 until end of experiment. Broilers were fed a wheat-soybean starter diet from d 0 to 10, a grower diet from d 10 to 21 and a finisher diet from d 21 to 39. The chronic cyclic heat stress model was implemented from d 24 till d 39. On d 28 to 31 increased mortality was noticed; in order to prevent excessive mortality, the high temperature during 6 h per day was decreased to 32 °C in period d 32 to 37 and to 33°C in period d 38 to 39.

**Supplementary Figure 2. (A)** Triiodothyronine (**T3**) and **(B)** T3/thyroxine (**T4**) in serum, and **(C)** glutathione in erythrocytes of male broilers subjected to chronic cyclic heat stress sampled at d 25 as affected by time after starting heat stress. Broilers were fed a wheat-soybean starter diet from d 0 to 10, a grower diet from d 10 to 21 and a finisher diet from d 21 to 39. The chronic cyclic heat stress model was implemented from d 24 till 39, i.e., the temperature was increased from 22 °C to 34 °C for 6 h per day (from 9:00 to 15:00) with relative air humidity (**RH**) between 52% and 58%. From 8:00 to 9:00, the temperature was increasing from 22 °C to 34 °C, and from 15:00 to 16:00, the temperature went down from 34 °C to 22 °C. The rest of the day the temperature was 22°C. On d 25, 1 bird per pen with a weight close to the average weight of the pen was selected. Sampling started minimum 3 h (180 min) after inducing heat stress.
